# Neurotoxicity induced by zinc oxide nanoparticles: age-related differences and interaction

**DOI:** 10.1038/srep16117

**Published:** 2015-11-03

**Authors:** Lei Tian, Bencheng Lin, Lei Wu, Kang Li, Huanliang Liu, Jun Yan, Xiaohua Liu, Zhuge Xi

**Affiliations:** 1 Department of Toxicology, Tianjin Institute of Health and Environmental Medicine, Tianjin, China

## Abstract

This study mainly investigated the neurotoxicity induced by zinc oxide nanoparticle (ZnO NP) in different-aged mice and the interaction between age and ZnO NP exposure. Sixty adult and old male C57BL/6J mice were assigned to four groups based on a two-factor (age and ZnO NP exposure) design. Results showed that ZnO NPs (5.6 mg/kg, intraperitoneal) induced increased production of pro-inflammatory cytokines in the serum and the brain of mice. A synergistic reaction between aging and ZnO NP exposure occurred regarding serum interleukin 1 (IL-1) and interleukin 6 (IL-6). In the brain, increased oxidative stress level, impaired learning and memory abilities, and hippocampal pathological changes were identified, especially in old mice, following ZnO NP exposure. Then, a potential mechanism of cognitive impairment was examined. The contents of hippocampal cAMP response element binding protein (CREB), phosphorylated CREB, synapsin I, and cAMP were decreased in an age-dependent manner, and the most substantial decrease occurred in old mice treated with ZnO NPs. These findings demonstrated for the first time that aging and ZnO NP exposure synergistically influenced systemic inflammation, and indicated old individuals were more susceptible to ZnO NP-induced neurotoxicity. One of the mechanisms might due to the supression of cAMP/CREB signaling.

Zinc oxide nanoparticles (ZnO NPs) are widely utilized in many products, such as toothpaste, beauty agents, sunscreens, textiles, wall paints, and building materials. Because of their special properties including small size and high specific surface area, the biological safety of nanomaterials has received wide attention. Toxicity of ZnO NPs has been extensively studied in different animal systems and cell types. Results showed that ZnO NPs could reach various organs after systemic distribution, and manifest hazardous effects on the lungs, liver, kidney, stomach, pancreas, spleen, testis, thymus, heart, brain and blood^1^,^2^,^3^,^4^,^5^. In addition, the cytotoxicities have also been found in many cultured cells such as epidermal cells^6^, macrophages^7^, human lung epithelial cells^8^, CHO cells^9^ and vascular endothelial cells^10^. Furthermore, ZnO NPs have been regarded as a possible treatment for cancer and autoimmune diseases^11^.

In recent years, more and more researches have provided evidence that NPs can reach the brain via blood-brain barrier (BBB) penetration or translocation along the olfactory nerve pathway and subsequently cause damage by the induction of oxidative stress, inflammatory responses, and cytotoxicity^12^. It has also been found that ZnO NPs could reach the brain after oral and inhalatrory administration in animals^13^,^14^, induce the changes in the spatial learning and memory ability of rats by altering the synaptic plasticity^15^, and interact diversely with plasma and brain proteins during inducing their toxic effects in the blood and brain^16^. *In vitro* studies, ZnO NPs showed the strongest toxicity for mice brain tumor cell lines (Neuro-2a) when compared with similar sized particles of Al_2_O_3_, TiO_2_, Fe_3_O_4_ and CrO_3_^17^, decreased the activity of mice neural stem cells and human SHSY5Y neuronal cells by inducing DNA damage and cell apoptosis^18^,^19^, and induced apoptosis in primary cultured astrocytes through JNK signaling pathway^20^. As we have known, these kinds of changes are involved in the onset and progression of neurodegeneration.

Neurodegenerative diseases belong to one class of age-related, slowly progressing neurological diseases that include Alzheimer’s disease (AD), Parkinson’s disease (PD), Huntington’s disease, and amyotrophic lateral sclerosis (ALS). In recent years, an increasing number of investigations have indicated that particulate matter (PM) and NPs in the environment may represent important risk factors for neurodegenerative diseases^21^. Several studies and reviews regarding the effects of air pollution on neurocognitive function, mood disorders, and CNS disease in child and adult populations have been published^22^,^23^,^24^. Other studies have also demonstrated an increased susceptibility to respiratory diseases and an increase in mortality in air polluted environments in individuals >65 years of age^25^,^26^,^27^.

As a result of the substantial amount of production and usage of ZnO NPs, it is necessary to evaluate their potential toxic effects on the CNS. Moreover, investigations regarding the possibility of susceptible subgroups are also critical to better understand the neurotoxicity of ZnO NPs, as well as to guide exposure standards. However, to date, whether an interaction exists between age and the potential adverse effects of ZnO NP exposure, as well as the exact mechanism have not been investigated. In this study, we aimed to determine the interaction between age and ZnO NP exposure and investigate the effects of ZnO NPs on cognitive function and the neural pathways involved in the mouse hippocampus.

## Results

### Characterization of ZnO NPs

**Figure 1:**
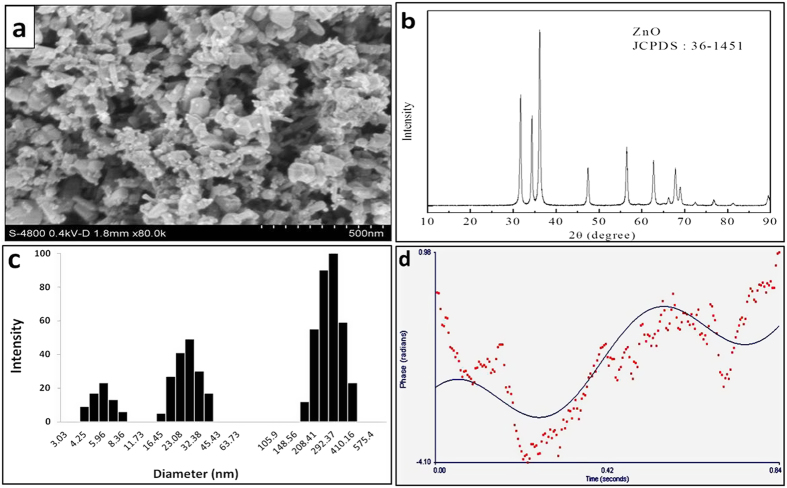
Characterization of ZnO NPs. (**a**) SEM image. (**b**) XRD pattern. DLS measurement of ZnO NPs for hydrodynamic size (**c**) and zeta potential (**d**).

Although the data available from the manufacturer indicated that the primary particle size of the ZnO NPs was <50 nm (Brunauer-Emmett-Teller method), the characterization of ZnO NPs was also detected in our laboratory. The SEM images demonstrated that ZnO NPs were within 20–80 nm in size, and the majority of the particles comprised a polygonal shape with smooth surfaces (Fig. 1a).The crystal structure of the ZnO NPs was characterized by XRD with Cu Kα radiation (λ = 0.15418 nm). Figure 1b shows the XRD patterns of the ZnO NPs. The peaks at 2θ=31.67°, 34.31°, 36.14°, 47.40°, 56.52°, 62.73°, 66.28°, 67.91°, 69.03°, and 72.48° were assigned to (100), (002), (101), (102), (110), (103), (200), (112), (201), and (004) of ZnO NPs, which indicates that the samples comprised a polycrystalline wurtzite structure (Zincite, JCPDS 36–1451). No characteristic impurity peaks were identified, which suggests a high quality of the ZnO NPs.

The sizes of the particles and agglomerates in normal saline were measured via DLS. As shown in Fig. 1c, with the exception of small size particles (diameter <65 nm), agglomerates formed in normal saline and were approximately ten times larger than the primary particle sizes of the ZnO NPs. The zeta potential was 6.01 mV for ZnO NPs suspended in MilliQ water (Fig. 1d).

### Systemic inflammation cytokines induced by ZnO NPs in different-aged mice

**Figure 2:**
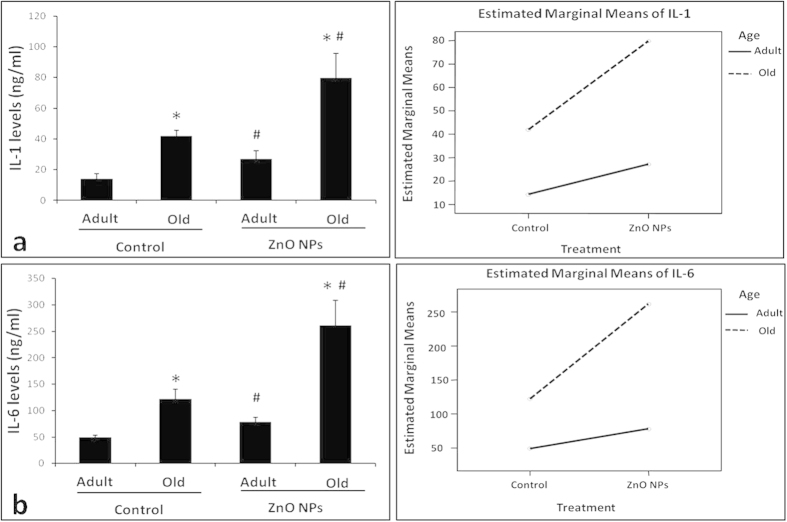
Influence of age and ZnO NP exposure on the serum levels of inflammatory factors. The left panels of (**a**,**b)** indicate the serum IL-1 and IL-6 levels in the adult and old mice after treatment with normal saline (the adult control group (AC) and the old control group (OC)) or ZnO NPs (the adult exposure group (AE) and the old exposure group (OE)) via i.p. In the right panels of (**a**,**b)**, the interaction diagrams demonstrate the synergistic effect between age and ZnO NP exposure. The data represent the mean ± SD, n = 5. *P < 0.05 vs. Adult group with the same treatment, and ^#^P<0.05 vs. Control group with the same age.

The serum levels of inflammatory cytokines in different groups of mice were measured as indicators of systemic inflammation and are shown in Fig. 2. Factorial analysis indicated that both age and ZnO NP exposure contributed to the increase in the serum inflammatory factors (P<0.05). ZnO NP exposure significantly increased the serum IL-1 and IL-6 levels in both the adult exposure group (AE) and the old exposure group (OE) groups compared with the respective control groups of the same age: the adult control group (AC) and the old control group (OC) (Fig. 2a,b, left panel, P<0.05). Moreover, increasing age (from adult to old) also significantly increased the serum IL-1 and IL-6 levels in the mice regardless of ZnO NP treatment (Fig. 2a,b, left panel). Interestingly, there was a synergistic reaction between age and ZnO NP exposure (P<0.05), and the serum IL-1 and IL-6 contents exhibited the highest level in the ZnO NP exposed old mice (Fig. 2, right panel).

### Brain inflammation cytokines induced by ZnO NPs in different-aged mice

**Figure 3:**
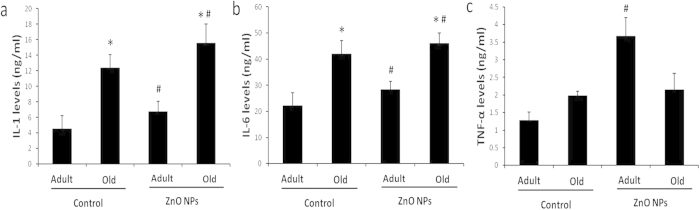
Influence of age and ZnO NP exposure on inflammatory factor levels in the brain. The brain IL-1 and IL-6 levels in the adult exposure group (AE) and the old exposure group (OE) were compared with the respective control groups: (the adult control group (AC) and the old control group (OC)) after 12 intraperitoneal injections of ZnO NPs. (**a**) IL-1 level. (**b)** IL-6 level. (**c**) TNF-α level. The data represent the mean ± SD, n = 5. *P<0.05 vs. Adult group with the same treatment, and ^#^P < 0.05 vs. Control group with the same age.

The IL-1, IL-6 and TNF-α level in the brain homogenate were measured. Figure 3a,b indicate that the IL-1 and IL-6 levels in the AE and OE groups were higher than the AC and OC groups, respectively (P<0.05), and the levels in the old mouse brains were also higher than the adult mouse brains in both the control and ZnO NP exposure groups (P<0.05). However, a distinct trend was identified in the TNF-α concentration. ZnO NP exposure significantly increased the TNF-α concentrations only in the adult mice compared with the control mice (Fig. 3c, P<0.05). There was no significant difference between the control and ZnO NP exposure groups in the old mice (P>0.05). No interaction between age and ZnO NP exposure was identified for the inflammatory reaction in the brain (P>0.05).

### Oxidative stress in the brain induced by ZnO NPs in different-aged mice

**Figure 4:**
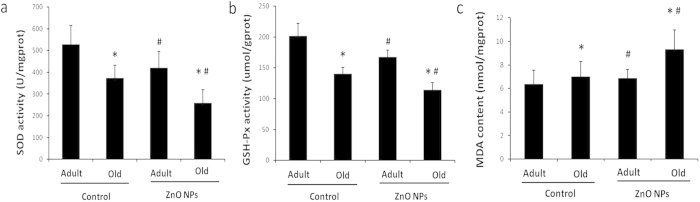
Effects of age and ZnO NP exposure on oxidative stress-related biomarkers in the brain. The brain oxidative stress-related biomarker levels in the adult exposure group (AE) and the old exposure group (OE) were compared with the respective control groups: (the adult control group (AC) and the old control group (OC)) after 12 intraperitoneal injections of ZnO NPs. (**a**) SOD activity. (**b**) GSH-Px activity. (**c**) MDA content. The data represent the mean ± SD, n = 5. *P<0.05 vs. Adult group with the same treatment, and ^#^P<0.05 vs. Control group with the same age.

MDA is the most representative product of lipid peroxidation, and its concentration can indicate the rate and intensity of lipid peroxidation within the body. SOD and GSH-Px concentrations directly reflect the antioxidant levels of the body. In Fig. 4a, the SOD concentrations in the AE and OE groups (420.67 ± 75.38 and 258.35 ± 61.58, respectively) were significantly decreased compared with the respective control groups: AC and OC (527.89 ± 87.08 and 372.99 ± 60.54, respectively, P<0.05). A similar trend was identified for the GSH-Px concentrations as shown in Fig. 4b: the ZnO NP exposure groups of adult and old mice (167.27 ± 11.74 and 114.21 ± 11.86, respectively) were significantly decreased compared with the respective control groups (201.68 ± 20.66 and 140.14 ± 10.97, respectively, P<0.05). The most significant increases in MDA content were identified in the brain homogenates of the old mice after ZnO NP exposure (P<0.05, Fig. 4c). Moreover, no interaction between age and ZnO NP exposure was identified for the oxidative stress reaction in the brain (P>0.05).

### Cognition function changes induced by ZnO NPs in different-aged mice

There were no significant differences in locomotor activity or exploratory behavior induced by ZnO NP exposure in the adult or old mice (data not shown) in the OFT. Learning and memory behavior were subsequently detected in the passive avoidance test (Table 1). The latency of the OE group (157.13 ± 25.9) was significantly longer compared with the OC mice (121.75 ± 29.2, P<0.05), whereas there was no significant difference between the AC and AE groups (265.0 ± 33.2 and 238.6 ± 26.0, respectively, P>0.05). The number of errors in both the OE and AE groups (5.63 ± 1.4 and 1.75 ± 0.7, respectively) were significantly greater than the OC and AC groups (2.50 ± 0.5 and 1.13 ± 0.6, respectively, P<0.05). These results suggest ZnO NP exposure impairs learning and memory behavior in mice, and more substantial memory loss was identified in the old mice.

### Alterations in hippocampal pathology in different-aged mice after ZnO NP treatment

**Figure 5:**
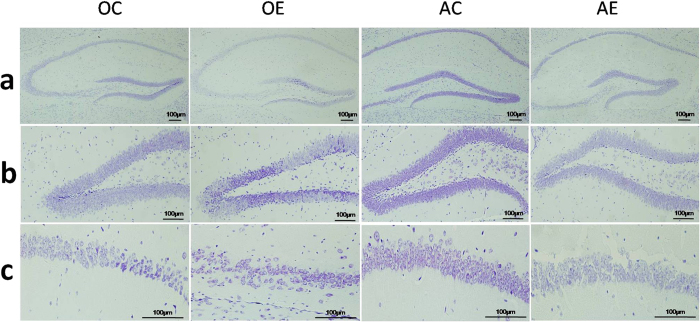
Changes in hippocampal pathology after 12 intraperitoneal injections of ZnO NPs. (**a**) Panorama gram of hippocampus, ×100. (**b**) Granular neurons in the hippocampal dentate gyrus (DG) region, ×200. (**c**) Pyramidal neurons in the hippocampal CA1 region, ×400. AC: the adult control group; AE: the adult exposure group; OC: the old control group and OE: the old exposure group.

Nissl staining is a widely used method to observe changes in neuronal morphology and pathology. This method specifically stains the cytoplasm of neurons in the brain. As illustrated in Fig. 5a, compared with the AC group, the hippocampal neurons and cytoplasmic Nissl bodies decreased obviously in the other three groups. The CA1 region is the most sensitive hippocampal region to oxidative damage; thus, it is also referred to as the area of vulnerability. The granular cell layer of the dentate gyrus (DG) region is involved in the formation of learning and memory^28^. Figure 5b,c indicated that neurons in the DG and CA1 region of the hippocampus in the AC group arranged closely and had abundant Nissl bodies in the cytoplasm. In the other three groups, pyramidal neurons in the hippocampal DG and CA1 regions were sparsely arranged, disordered, and contained dissolved and fewer Nissl bodies. The interaction between age and ZnO NPs resulted in more serious nerve tissue injury in the DG and CA1 hippocampal regions in the OE group compared with the AE group. In the OE group, the pathology indicated thin Cresyl violet staining in the cytoplasm, and the Nissl bodies were dramatically decreased in number or even absent in neurons. The cellular gaps increased, part of the cells were vacuolated, and irregular morphologies were present (Fig. 5b,c).

### Hippocampal CREB activity changes in different-aged mice after ZnO NP treatment

**Figure 6:**
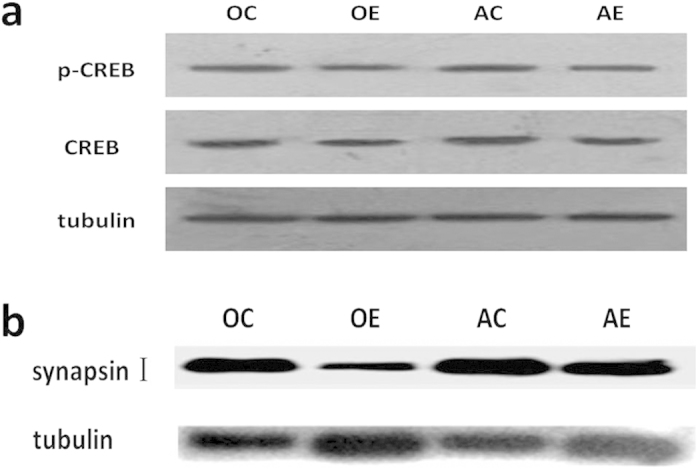
Hippocampal protein expression changes in different-aged mice after ZnO NP exposure. (**a**) Hippocampal CREB and p-CREB expression levels in four different groups of mice (AC: the adult control group; AE: the adult exposure group; OC: the old control group and OE: the old exposure group). Proteins and pre-stained protein marker were transferred from the gel to PVDF membrane using a tank system at 300 mA for 60 min. Then, the membrane was cut into 3 parts according the pre-stained protein marker: (1) at the position of the protein marker between 50 KDa (red band) and 40 KDa (blue band); (2) at the position of the protein marker below 30 KDa (blue band). The up part of the membrane with protein MW≥50 KDa was used to detect the expression of tubulin (loading control). The middle part of the membrane with protein MW between 30 KDa and 50 KDa was used to detect the p-CREB expression firstly, and then was stripped and reprobed with CREB. The images displayed are cropped (full-length blots are presented in Supplementary Figure S1). (**b)** Hippocampal synapsin I expression levels in the four groups of mice. Proteins and pre-stained protein marker were transferred from the gel to PVDF membrane using a tank system at 300 mA for 90 min. Then, the membrane was cut at the position of the protein marker between 50 KDa (red band) and 40 KDa (blue band). The up part of the membrane with protein MW≥50 KDa was used to detect the expression of synapsin I firstly, and then was stripped and reprobed with tubulin as a loading control. The images displayed are cropped (full-length blots are presented in Supplementary Figure S2). Samples are derived from the same experiment and that blots were processed in parallel. Cropped pictures are shown.

CREB plays a central role in memory formation and synaptic plasticity, and its activation is controlled by phosphorylation modification at the Ser133 site^29^. Therefore, the expression changes of CREB and p-CREB in the hippocampus after ZnO NP treatment were measured via western blotting and are shown in Fig. 6a. The CREB levels in the OE and AE groups were significantly reduced compared with their respective control groups (equivalent to 71.87% of the OC group and 69.86% of the AC group, P<0.05). The CREB levels in the old mice were significantly decreased compared with the adult mice that received the same treatment (P<0.05). Furthermore, the hippocampal p-CREB levels in the different-aged mice after ZnO NP treatment were also significantly reduced compared with their respective control groups (equivalent to 41.92% of the OC group and 54.91% of the AC group, respectively; P<0.01). The difference in the p-CREB levels between the adult and old mice that underwent the same treatment was not significant, although a trend towards a decrease was identified in the hippocampi of the old mice (P=0.06). These data demonstrate that ZnO NPs and aging significantly affected hippocampal p-CREB expression.

Synapsin I is a major endogenous substrate for CREB and plays a key role in synapse formation and plasticity^30^. The changes in hippocampal synapsin I expression between the different-aged mice after ZnO NP treatment are shown in Fig. 6b. The synapsin I levels in the OE group were significantly reduced compared with the OC group (i.e., equivalent to 22.99% of the OC group; P<0.01). In the AE group, the synapsin I levels were also significantly lower (78.08%) than the AC group (P<0.05). The difference in the synapsin I levels between the adult and old mice that underwent the same treatment was also remarkable, with significantly decreased hippocampal expression in the old mice (P<0.05 vs. Control group, and P<0.01 vs. ZnO NP exposure group).

### Effect of ZnO NP exposure on the hippocampal cAMP content in different-aged mice

**Figure 7:**
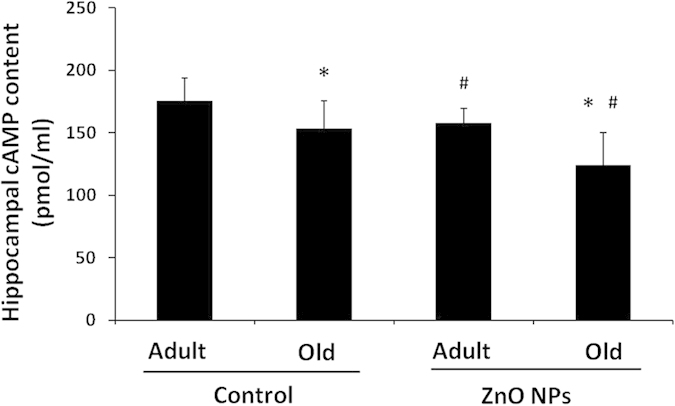
Changes in the hippocampal cAMP content in different-aged mice treated with ZnO NPs. Mice were divided into four groups (AC: the adult control group; AE: the adult exposure group; OC: the old control group and OE: the old exposure group).The data represent the mean ± SD, n = 5. *P<0.05 vs. Adult group with the same treatment, and ^#^P < 0.05 vs. Control group with the same age.

The alterations in the hippocampal cAMP content in the different-aged mice after ZnO NP treatment are shown in Fig. 7. The cAMP levels in the AE and OE groups (157.66 ± 12.14 and 124.40 ± 25.61, respectively) were significantly lower than the AC and OC groups (175.62 ± 18.21 and 153.68 ± 22.14, respectively; P<0.05). The cAMP level in the OE group was significantly decreased compared with the other three groups (P<0.05). There was no interaction between age and ZnO NP exposure (P>0.05).

## Discussion

In recent years, evidence has indicated that nanomaterials, including ZnO NPs, may cause toxic effects on the CNS, including cytotoxicity, genotoxicity, the induction of oxidative stress, and inflammation^31^. These adverse effects are also involved in the onset and progression of neurodegeneration^32^. Thus, the relationship between nanomaterial exposure and their capability to induce neurodegeneration has received substantial attention^33^.

Advanced age is the most important risk factor for the development of neurodegenerative disorders of the brain; thus, adult (6-month-old) and old (18-month-old) C57BL/6J male mice were utilized to characterize the interaction between age and ZnO NP exposure. According to the growth cycle of C57BL/6J mice, a 6-month-old mouse has an equivalent physiological age to a 30-year-old human, whereas an 18-month-old mouse is equivalent to a 56 year-old human in a healthy environment^34^. For individuals who live beyond the age of 60 years, both cognitive impairment and dementia become increasingly prevalent^35^.

First, we demonstrated that ZnO NPs induced a systemic inflammatory reaction in both adult and old mice. Moreover, age also contributed to systemic inflammation. A significant synergistic reaction between age and ZnO NP exposure for the serum IL-1 and IL-6 contents was identified. These data indicate that ZnO NP exposure not only induced a systemic inflammatory reaction but more serious systemic inflammation occurred with increasing age. It has been previously reported that serum pro-inflammatory mediators are increased after ZnO NPs exposure^1^ and up-regulated continuously during the aging process^36^,^37^. In the current study, the most significant increase in the serum inflammatory cytokine levels of the old mice was identified after ZnO NP exposure. Thus, an aging individual may exhibit more harmful disturbances during nanoparticle exposure, such as ZnO NPs.

Second, the neurotoxicity was identified in mice after administration of ZnO NPs by intraperitoneal injection. ZnO NP exposure induced increased production of pro-inflammatory cytokines in the brain. Although no synergistic reaction was found between age and ZnO NP exposure, the levels of pro inflammatory cytokines were highest in the old mice with ZnO NP exposure. Moreover, significantly decreased SOD and GSH-Px concentrations and an increased MDA concentration were identified in the brain homogenate of ZnO NP-treated mice. The most obvious changes were identified in the old mice treated with ZnO NPs. These data indicated that ZnO NPs exposure could induce inflammation reaction and oxidative stress in the CNS, and the same amount of ZnO NP exposure induced more exaggerated effects in the brains of old individuals. It has been well demonstrated that the induction of oxidative stress plays a critical role in the common mechanism of nanoparticle toxicity^38^. Prolonged and serious oxidative stress may cause an increase in inflammation via the activation of inflammation-related genes^39^.

The BBB is the interface between the vascular system and the brain, and is critical for regulating substances transport into the brain. Abundant evidences indicated that nanomaterials (including ZnO NPs) in the circulation could induce alterations in the integrity and permeability of BBB via the following mechanisms: oxidative stress on endothelial cells^40^,^41^, nanoparticle induced endothelial cell leakiness^42^,^43^,^44^, and inflammatory pathway^45^,^46^. And then, nanoparticles and/or pro-inflammatory mediators could enter the brain to cause toxic effects on the CNS. In this study, although the influence of ZnO NPs on BBB hasn’t been detected, we can speculate that the remarkable increased serum pro-inflammatory mediators induced by ZnO NPs and nanoparticles themselves might destroy BBB. Then, ZnO NPs can induce the neurotoxicity by direct enter into the brain and/or indirect systemic inflammatory effect. Besides, It has been well demonstrated that oxidative stress^47^ and inflammation^48^,^49^ are altered in aging. Our findings suggest that it might be easier to induce BBB destruction in old individuals after ZnO NPs exposure.

Third, the effects of ZnO NP exposure on neurocognitive function in adult and old mice were explored. Our data demonstrated that the long-term memory and passive avoidance ability of mice were impaired by ZnO NP treatment in different-aged mice, especially in the OE group. However, no significant changes in locomotor activity or exploratory behavior were identified. ZnO NPs have been demonstrated to damage spatial cognition capability via over-enhanced long-term potentiation in the hippocampus of Wistar rats^15^. However, Xie *et al.* reported that ZnO NPs ameliorated behavioral and cognitive impairments in Swiss male mice with depressive-like behaviors induced by LPS^50^. These authors demonstrated that ZnO NP treatment increased the numbers of crossings and rearings in the OFT; however, the treatment did not influence the target quadrant or number of platform crossings in the Morris water maze. This contradiction may be because of the different characterizations of ZnO NPs and the induction time. In Xie’s experiment, 5.6 mg/kg body weight of ZnO NPs was injected i.p. into mice every other day for eight injections, which may not be sufficiently toxic to cause cognitive impairment.

Substantial evidence suggests the hippocampus is closely related to cognitive ability, including learning and memory. In this study, we demonstrated that ZnO NPs induced cognitive deficiency and pathological changes in the hippocampus, and there were greater cognitive impairments in old mice compared with their adult counterparts. The hippocampus has been demonstrated to exhibit high constitutive expression of IL-1 receptors on neurons and glia cells in the granule cells of the DG and pyramidal cell layer^51^,^52^, as well as increased and faster expression of pro-inflammatory cytokines compared with other brain regions following a peripheral immune challenge^53^. We deduced that the adverse functional and structural alterations of the hippocampus may result, in part, from the systemic and CNS inflammation induced by ZnO NP exposure, and the old individuals may be more susceptible to ZnO NP-induced damage because of the synergistic reaction between age and ZnO NP exposure.

Finally, the mechanism that underlies ZnO NP-induced impairment of cognitive function was investigated. Long-term memory formation requires new gene transcription and subsequent new protein synthesis in the CNS. An increasing number of studies regarding organisms that range from invertebrates to mammals have suggested that CREB acts as the molecular switch that may represent the core component controlling long-term synaptic plasticity and long-term memory^54^,^55^. Thus, we examined the changes in cAMP/CREB signaling in the brain after ZnO NP exposure in different-aged mice. In the present study, the hippocampal CREB and p-CREB protein levels decreased in an age-dependent manner and were further down-regulated after ZnO NP treatment in both adult and old mice. As the main substrate of CREB, the hippocampal synapsin I expression exhibited a similar trend with the lowest level identified in the hippocampi of the ZnO NP-treated old mice. Synaptic plasticity of neurons includes functional plasticity and structural plasticity, which is closely related to learning and memory processes. Synapsin 1 is one of the important proteins involved in learning, memory and LTP (long-term potentiation). In this experiment, the expression of synapsin 1 in the hippocampus of mice was reduced by zinc oxide exposure and age, which was consistent with the changes in the behavior of the mice^56^,^57^. In addition, the hippocampal cAMP levels in the ZnO NP-treated old mice significantly decreased, which may result in abnormal signal transduction cascades. These findings strongly suggest that the influence of the cAMP/CREB signaling pathway by ZnO NPs and aging are involved in learning and memory impairment in C57BL/6J mice.

Nevertheless, there are several limitations regarding this study that should be considered. For example, we only investigated the effects of a single dose (5.6 mg/kg body weight) and one size of ZnO NPs, as well as a single treatment paradigm (12 treatments). Thus, the comparison of neurotoxicity between different sizes of ZnO NPs and chronic exposures at lower concentrations should be considered in future studies.

## Conclusions

ZnO NPs could induce a systemic inflammatory reaction in both adult and old mice, and there existed a synergistic reaction between age and ZnO NP exposure during inducing systemic proinflammation mediators. In the brain, the oxidative stress and inflammation reaction were found after ZnO NP exposure and more remarkable changes were identified in aged individual. These kinds of neurotoxicity might due to the destruction of the BBB integrity caused partly by ZnO NPs-induced systemic inflammation. In addition, ZnO NP exposure resulted in abnormal cognitive function and neuronal pathological changes in the hippocampus. Old mice exhibited greater susceptibility to ZnO NP-induced damage. The intrinsic molecular mechanism for the impairment of long-term memory may result, in part, from the repression of the cAMP/CREB pathway and the consequent decreases in hippocampal p-CREB and synapsin I levels. To the best of our knowledge, this is the first study to demonstrate that aging and ZnO NP exposure synergistically influence systemic inflammation. Furthermore, these findings provide clues for further investigation into the specific mechanism that underlies the effects of ZnO NPs on neurodegenerative diseases.

## Materials and Methods

### Characterization of ZnO NPs and particle suspension preparation

ZnO NPs were purchased from Sigma Aldrich (Saint Louis, MO, USA). Morphological analysis of single particles was performed using a field emission scanning electron microscope (SEM; FESEM, JSM-7600F, JEOL Ltd., Tokyo, Japan) equipped with Soft Imaging System. Particle dispersions were prepared and subsequently centrifuged onto glass cover slips. The particles were allowed to air dry on the cover slips prior to mounting on SEM stubs.

The crystalline nature of the prepared ZnO NPs was determined using the x-ray diffraction (XRD) pattern at 25–28 °C with a PANalytical X’Pert x-ray diffractometer (Mini Flex II, Rigaku Corporation, Tokyo, Japan), which was equipped with a nickel (Ni) filter. The x-ray source comprised Cu Kα (λ=1.54056 Å) radiation.

A dynamic light scattering (DLS) instrument (ZETAPALS/BI-200SM, Brookhaven, NY, USA) was used to measure the hydrodynamic size and zeta potential at 25 °C. Filtered normal saline and MilliQ water (0.2 μm) were used to prepare particles for measurement of the hydrodynamic size and zeta potential, respectively. Following sonification (ten times, 30 s every 2 min), the suspensions were immediately detected.

ZnO NPs used for animal exposure were prepared in normal saline by sonication (ten times, 30 s every 2 min) at 4 °C. The NPs were vortexed 5 times for 10 s prior to usage to break down agglomerates and ensure a uniform suspension.

### Animal treatment

Healthy male C57BL/6J mice were obtained from the Academy of Military Medical Sciences (Beijing, China). The animals were housed in an environmentally controlled room at 23 ± 1.5 °C with a 12-h light/12-h dark cycle. Six- (24.87 ± 1.50 g) and 18 (19.19 ± 0.88 g) month-old mice represented the adult and old animals, respectively. The mice were randomly divided into four groups (n = 15 per group): 1) the adult control group (AC); 2) the adult exposure group (AE); 3) the old control group (OC); and 4) the old exposure group (OE). Animal grouping was based on a two-factor cross-classification model: age and administration. The age factor involved two levels (adult/old) and the administration factor involved two conditions (control/exposure). C57BL/6 J mice were administered an intraperitoneal (i.p.) injection of ZnO NPs at 5.6 mg/kg body weight three times per week for four weeks. The control groups (AC and OC) were treated with an equivalent volume of normal saline. All animal experiments were performed in accordance with protocols approved by the National Animal Ethics Committee of China.

### Cognition function

The open field test (OFT) was used to assess both exploratory behavior and learning activity. The open field box was composed of wood with a 50 × 50 cm surface area and four walls 38 cm in height. The bottom was divided into 16 boxes, which included the peripheral lattice (12) and the central lattice (4). Each mouse was placed in the middle of the open field for a single 10 min session with the experimenter out of sight. The distance traveled and time spent in each area was recorded. After each trial, fecal boli were removed, and the floor was wiped with 70% ethanol and dried. The control (AC and OC) and experimental (AE and OE) mice were treated with normal saline or ZnO NPs, respectively, and subsequently examined in the OFT. Ten mice per group were subsequently selected randomly for the passive avoidance test.

The shuttle box comprised two identical compartments (50 × 16 × 18 cm) divided into bright and dark rooms. The bottom of the box was composed of stainless steel rods with 36 volts and 50 HZ alternating current, with animal excrement collectors located underneath. An interface was connected to the computer to automatically collect and record data.

### Inflammatory and oxidative stress level assay

After the final treatment, the C57BL/6 J mice were anesthetized with chloral hydrate (300 mg/kg, i.p.), and blood samples were collected through heart puncture without an anticoagulant. After 30 min at room temperature, the blood samples were centrifuged at 2000 × g for 15 min at 4 °C. The serum was subsequently transferred to clean test tubes and stored at −80 °C.

After the mice were euthanized, fresh brain tissues were excised and weighed. A 1:9 (w/v) volume of ice-cold physiological saline was added, and the mixtures were homogenized in an ice bath. The homogenates were centrifuged at 3000 rpm for 10 min at 4 °C. The supernatants were collected and assayed for oxidative biomarkers. The protein contents were determined using the Bradford method with bovine serum albumin (BSA) as the standard substance (Beyotime Biotechnology, Haimen, China).

Interleukin 1 (IL-1), interleukin 6 (IL-6), and tumor necrosis factor alpha (TNF-α) levels were measured according to the manufacturer’s instructions using a double-antibody sandwich enzyme linked immunosorbent assay (ELISA) kit (R&D Systems, minneapolis, MN, USA). Glutathione peroxidase (GSH-Px) and superoxide dismutase (SOD) activities and the level of the lipid peroxidation product malondialdehyde (MDA) were determined using commercial kits from Nanjing Jiancheng Bioengineering (Nanjing, China). The optical density was measured at 450 nm using an Automatic Multi-function Microplate Reader (Multiskan MK3; Thermo Scientific, Waltham, MA, USA).

### Tissue processing for histology

For histological examination, mice from each group (n = 6) were deeply anesthetized with chloral hydrate (300 mg/kg, i.p.) and transcardially perfused with 0.1 M phosphate-buffered saline (PBS, pH 7.4) followed by 4% paraformaldehyde (PFA) in 0.1 M phosphate buffer (PB, pH 7.4). Following perfusion, the brains were carefully dissected, post-fixed in formalin for 12 h, and cryoprotected by infiltration with 30% sucrose for 3 days (d) at 4 °C. After dehydration, the brains were embedded with optimal cutting temperature (OCT) compound, and serial coronal sections 8 μm in thickness were collected in the horizontal plane using a freezing microtome (CM1900, Leica, Nussloch, Germany).

### Nissl staining

To assess the histopathological changes, the sections were further subjected to Nissl staining using a well-established protocol^58^. In brief, the slides were immersed in 0.1% cresyl violet at room temperature for 10 min. After rinsing with distilled water, the sections were dehydrated in graded alcohol (70%, 95%, and 100%, 2 × ), placed in xylene, cover slipped with neutral balsam, and examined with a light microscope. Six brain sections were selected per group (n = 6).

### Western blotting

Hippocampal tissues were harvested and homogenated with RIPA lysis buffer on crushed ice. Following 20 min of incubation, the insoluble material was removed by centrifugation at 10,000 × g for 20 min at 4 °C. The protein content was determined with a bicinchoninic acid (BCA) protein assay kit (Nanjing Jiancheng Bioengineering Institute, Nanjing, China), and 40 μg of total protein were loaded in each lane, subjected to electrophoresis, and subsequently transferred to polyvinyl difluoride (PVDF) membranes (Millipore Corp., Bedford, MA, USA) via electroblotting. The membranes were blocked in PBS buffer that contained 0.05% Tween-20 and 5% milk for 1 h at 37 °C and then incubated with a rabbit monoclonal antibodies from Abcam plc (Cambridge, UK) against mouse cAMP response element binding protein (CREB), phospho-CREB (p-CREB; S133) and tubulin, and rabbit polyclonal antibody from BOSTER (Wuhan, China) against synapsin I in blocking solution overnight at 4 °C. After washing twice with PBST, immunoreactive proteins in the membrane were visualized using enhanced chemiluminescence (ECL) reagents (Santa Cruz, Dallas, Texas, USA) with a horseradish peroxidase-conjugated secondary antibody (1:5000) (Santa Cruz, Dallas, Texas, USA). The chemiluminescence results were scanned and densitometrically analyzed using an ImageMaster VDS system (Amersham, UK) with an Imagequant TL site program.

### Hippocampal cAMP content

After the mice were euthanized, the hippocampi were separated from the brain, frozen in liquid nitrogen, and then ground to a fine powder under liquid nitrogen in a stainless steel mortar. When the liquid nitrogen evaporated, the frozen tissues were weighed and homogenized in 10 volumes of 0.1 M hydrochloric acid (HCl). After centrifugation at 600 × g at room temperature, the samples were diluted in 0.1 M HCl, and detection was conducted using a cAMP Enzyme Immunoassay Kit from Sigma Aldrich (Saint Louis, MO, USA) as described in the manufacturer’s instructions.

### Statistical analysis

The data are represented as the mean ± standard deviation (SD). The interaction between age and treatment was first analyzed via variance analysis of the two-factor two-level factorial design. If there was no interaction between two factors, one-way analysis of variance (ANOVA) followed by a post-hoc Dunnett’s test was used to evaluate group differences. The statistical significance for all tests was set at P<0.05. All data were analyzed using SPSS 16.0 (SPSS, Inc., Chicago, IL, USA).

## Additional Information

**How to cite this article**: Tian, L. *et al.* Neurotoxicity induced by zinc oxide nanoparticles: age-related differences and interaction. *Sci. Rep.* **5**, 16117; doi: 10.1038/srep16117 (2015).

## Supplementary Material

Supplementary Information

## Figures and Tables

**Table 1:** Changes in the latency and number of errors in different-aged mice after ZnO NPs treatment.

Groups	Animal number	Latency (s)	Number of errors
AC	10	265.0 ± 33.2	1.13 ± 0.6
AE	10	238.6 ± 26.0	1.75 ± 0.7^#^
OC	10	121.75 ± 29.2	2.50 ± 0.5
OE	10	157.13 ± 25.9^#^	5.63 ± 1.4^#^

Mice were divided into four groups (AC: the adult control group; AE: the adult exposure group; OC: the old control group and OE: the old exposure group). Each data represents mean ± SD. ^#^p<0.05 vs Control group with the same age.
