# Supplementary material for: Neurotoxicity induced by zinc oxide nanoparticles: age-related differences and interaction
Source: Sci Rep. 2015 Nov 3;5:16117. doi: 10.1038/srep16117 (PMC4630782; doi:10.1038/srep16117)
Supplement: Supplementary Information [file srep16117-s1.doc]

**Supplementary information:**

Neurotoxicity induced by zinc oxide nanoparticles: age-related differences and interaction

Lei Tian, Bencheng Lin, Lei Wu, Kang Li, Huanliang Liu, Jun Yan, Xiaohua Liu, and Zhuge Xi

**Supplementary Figure S1**

**
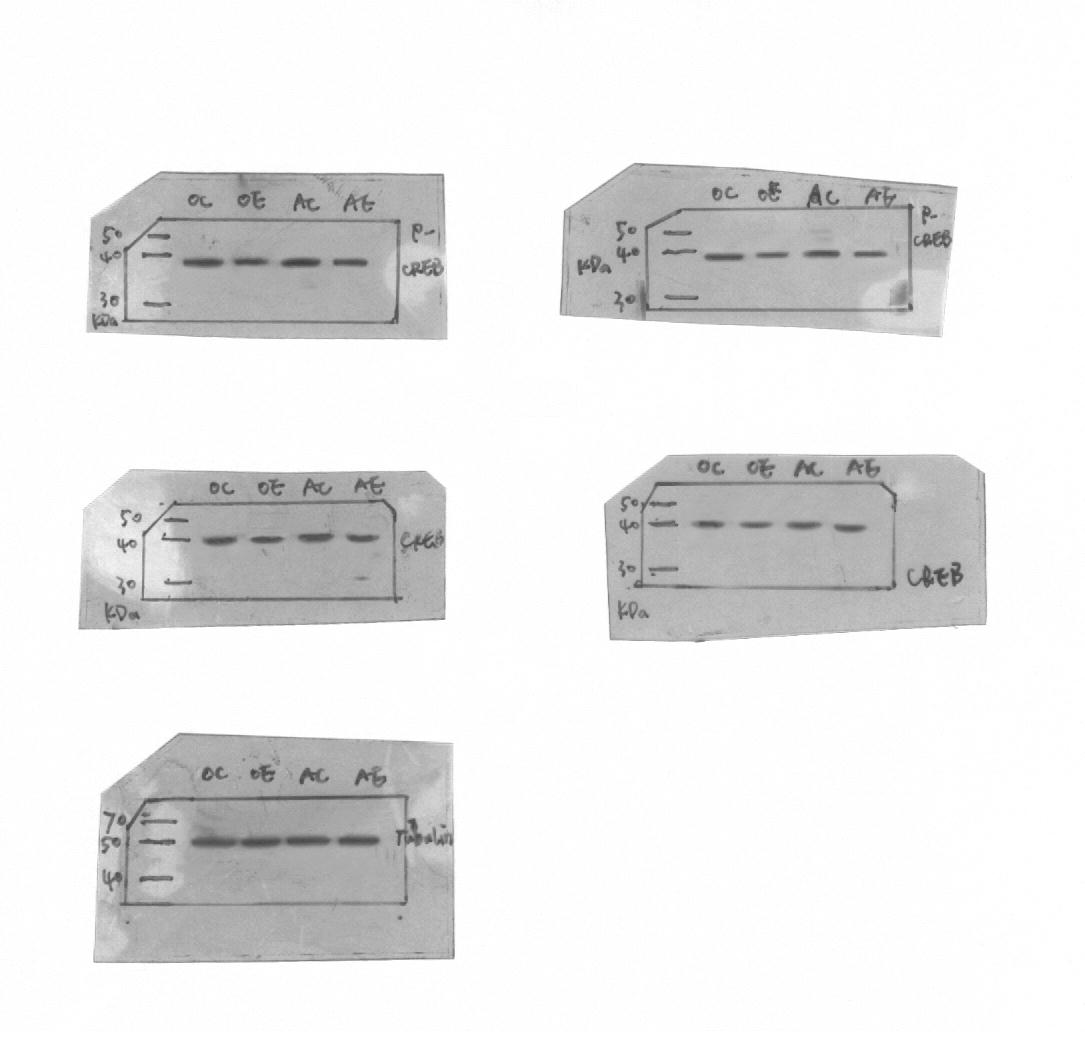
**

a


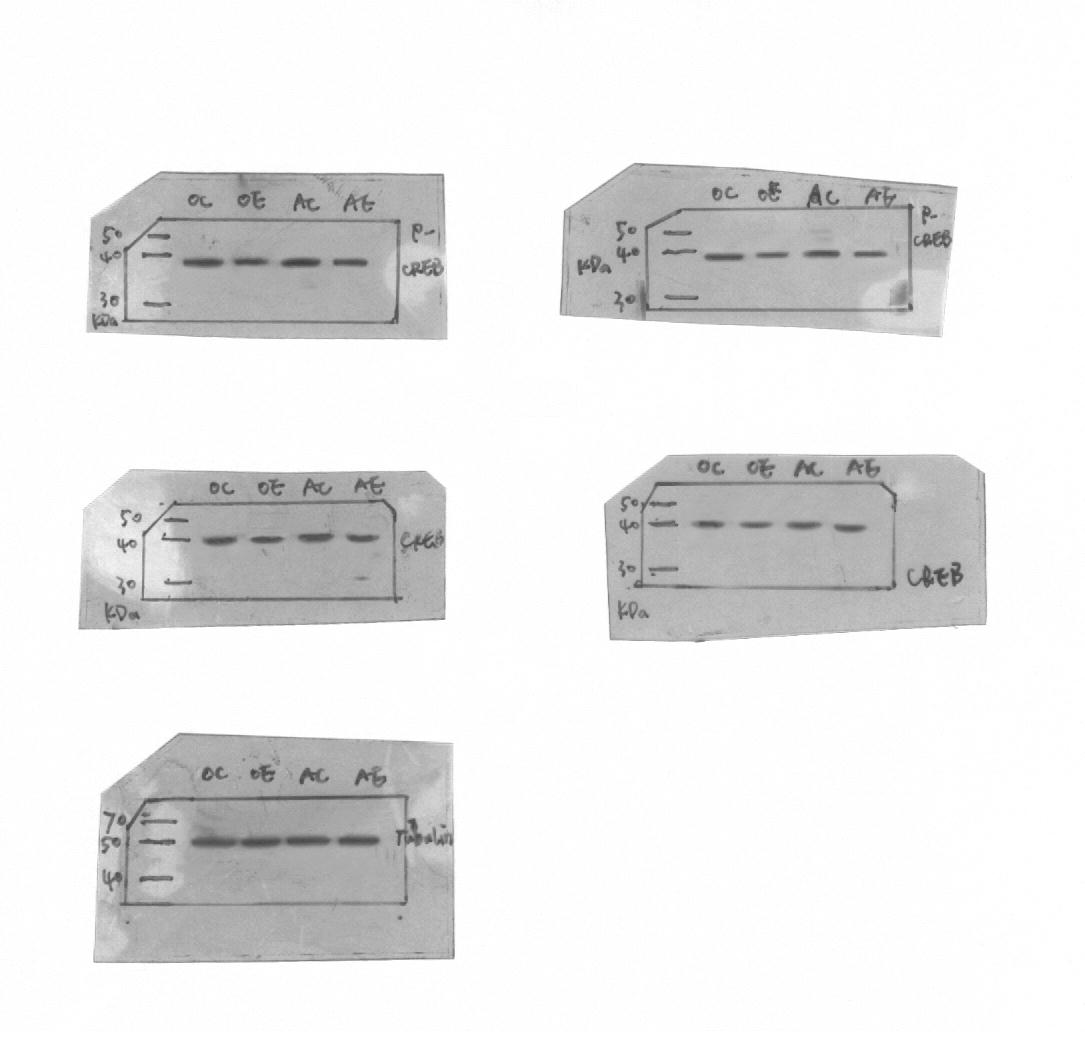


b


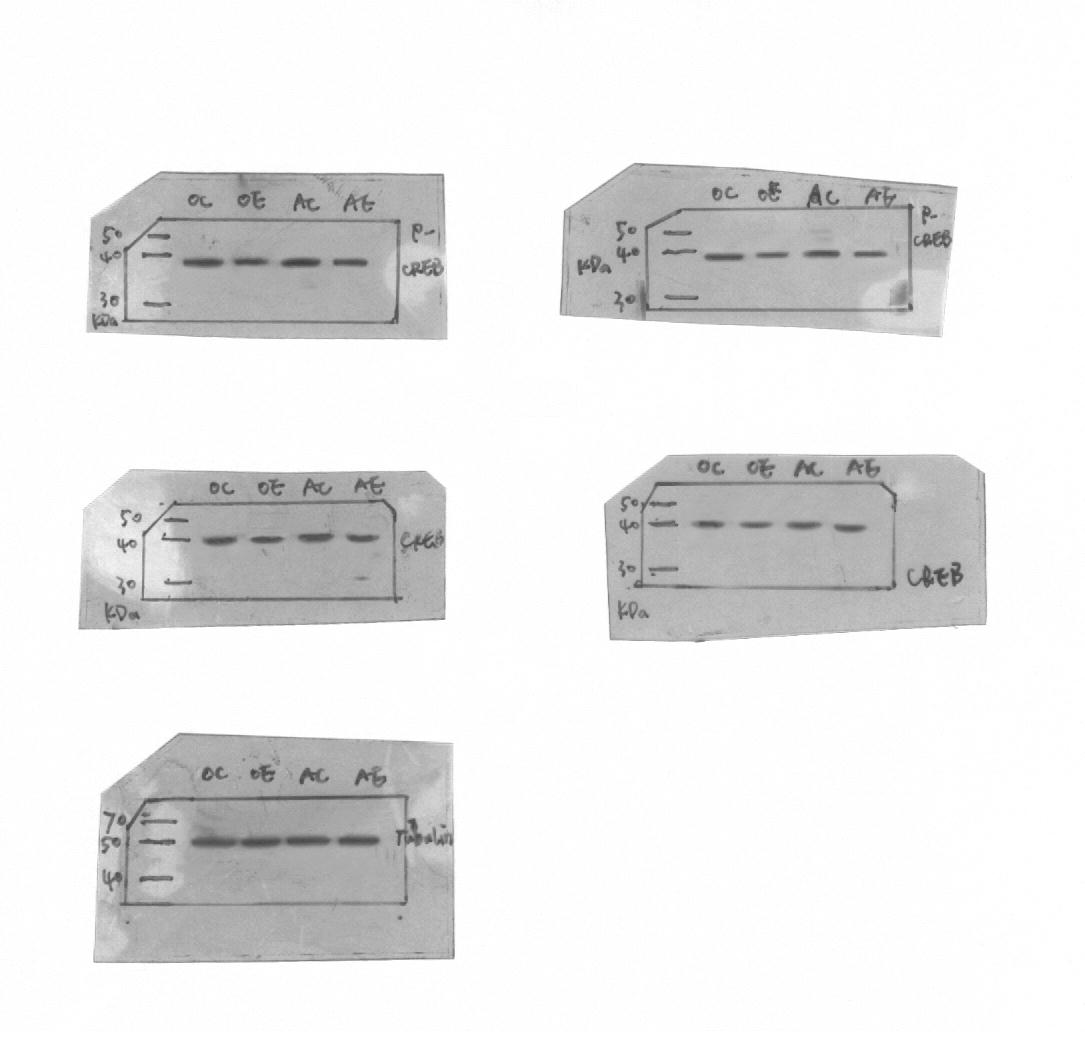


c

**Supplementary Figure S1. Western blot analysis of hippocampal CREB and p-CREB expression in the mice.** Hippocampal proteins were collected from four different groups of mice. Afer SDS-PAGE, proteins were transferred from the gel to PVDF membrane using a tank system at 300 mA for 60 min. Films with different exposure time were showed. **(a)** The middle part of the membrane with protein MW between 30 KDa and 50 KDa was firstly used to detect the p-CREB expression. (b) The same membrane was stripped and reprobed with CREB. (c) The up part of the membrane with protein MW≥50 KDa was used to detect the expression of tubulin (loading control).

**Supplementary Figure S2**

**
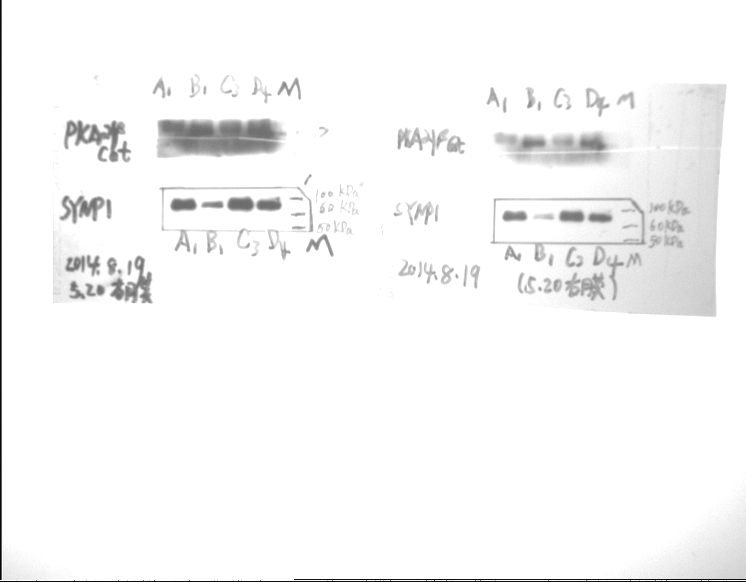

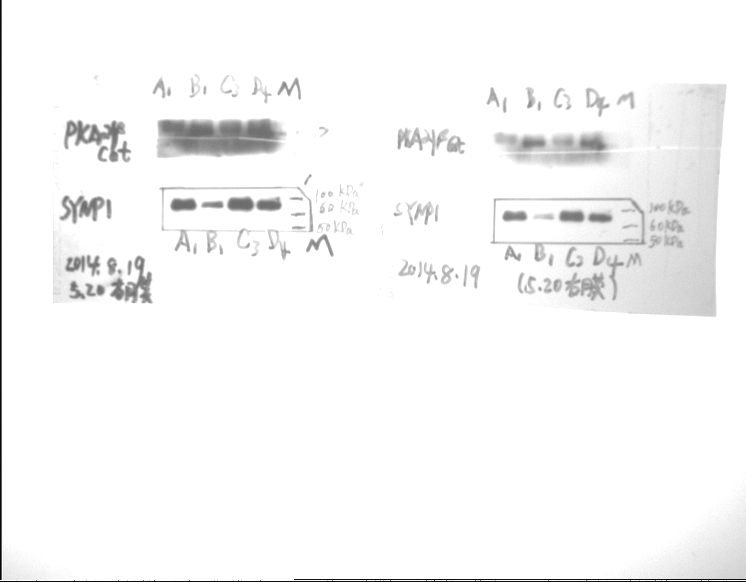
**

a


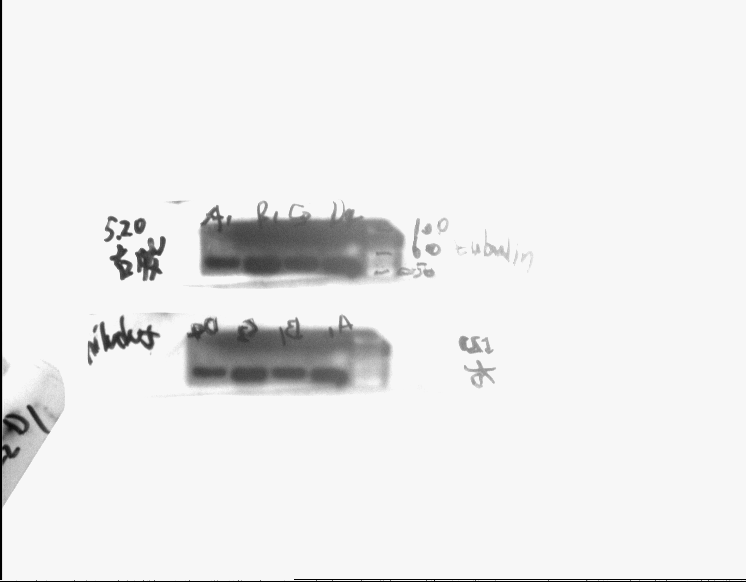


b

**Supplementary Figure S2. Western blot analysis of hippocampal synapsin I expression in the mice.** Hippocampal proteins were collected from four different groups of mice. Afer SDS-PAGE, proteins were transferred from the gel to PVDF membrane using a tank system at 300 mA for 90 min. Films with different exposure time were showed. **(a)** The up part of the membrane with protein MW≥50 KDa was firstly used to detect the expression of synapsin I. (b) The same membrane was stripped and reprobed with tubulin (loading control). A represents OC grouup, B represents OE grouup, C represents AC grouup, and D represents AE grouup.
